# Overcoming challenges in conducting systematic reviews in implementation science: a methods commentary

**DOI:** 10.1186/s13643-023-02285-3

**Published:** 2023-07-07

**Authors:** Anna Chapman, Nicole M. Rankin, Hannah Jongebloed, Sze Lin Yoong, Victoria White, Patricia M. Livingston, Alison M. Hutchinson, Anna Ugalde

**Affiliations:** 1 Centre for Quality and Patient Safety Research, School of Nursing and Midwifery, Faculty of Health, Institute for Health Transformation, Deakin University, Geelong, VIC Australia; 2 School of Population and Global Health, Centre for Health Policy, University of Melbourne, Melbourne, VIC Australia; 3 Global Centre for Preventive Health and Nutrition, School of Health and Social Development, Faculty of Health, Institute for Health Transformation, Deakin University, Geelong, VIC Australia; 4 School of Psychology, Faculty of Health, Deakin University, Geelong, VIC Australia; 5 Barwon Health, Geelong, VIC Australia

**Keywords:** Implementation science, Systematic review, Implementation, Dissemination, Knowledge translation

## Abstract

Consolidation of the literature using systematic reviews is a critical way to advance a discipline and support evidence-based decision-making in healthcare. However, unique challenges exist that impact the conduct of systematic reviews in implementation science. In this commentary, we reflect on our combined experience to describe five key challenges unique to systematic reviews of primary implementation research. These challenges include (1) descriptors used in implementation science publications, (2) distinction between evidence-based interventions and implementation strategies, (3) assessment of external validity, (4) synthesis of implementation studies with substantial clinical and methodological diversity, and (5) variability in defining implementation ‘success’. We outline possible solutions and highlight resources that can be used by authors of primary implementation research, as well as systematic review and editorial teams, to overcome the identified challenges and optimise the utility of future systematic reviews in implementation science.

## Background

Implementation science seeks to improve the quality and safety of healthcare through the scientific study of strategies that promote the integration of evidence-based interventions and/or policies into real-world settings [1]. The discipline aims to reduce the substantial time lag between the creation of evidence and its widespread uptake, and consequently, policy-makers and funding bodies are increasingly prioritising investment in implementation research [2]. A vast and growing body of primary implementation research now exists, and efforts to consolidate the available literature has resulted in a proliferation of systematic reviews in implementation science (as evidenced by the large number of reviews previously published by the Cochrane Effective Practice and Organisation of Care (EPOC) group, and within implementation science journals).

Systematic reviews are a rigorous and robust approach to identify, appraise, and synthesise findings from primary research studies. They are considered ‘gold standard’ because they provide cumulative evidence to guide evidence-based decision-making that addresses spurious findings from individual research studies [3]. The scope of systematic reviews of implementation studies in healthcare can include, but is not limited to, collating and evaluating measurement tools/instruments [4]; understanding the applicability of theories, models and/or frameworks [5]; exploring barriers and facilitators to implementation [6]; investigating potential for implementation [7]; examining the use/effectiveness of implementation strategies [8]; understanding the mechanisms of implementation strategies [9]; and examining constructs that influence implementation (e.g. context [10], organisational readiness [11]).

The findings of systematic reviews of primary implementation research have the capacity to advance the discipline and optimise healthcare. Yet, review teams commonly face challenges when identifying, appraising, and synthesising primary implementation research. While standards exist to guide the transparent and accurate reporting of primary implementation studies (Standards for Reporting Implementation Studies (StaRI) [12] checklist), guidance does not extend to systematic reviews of implementation research (such as a PRISMA extension). By optimising the utility of future systematic reviews, the strength and applicability of review findings to real-world contexts and problems will be enhanced. In this commentary, we reflect on our combined experience of conducting systematic reviews in the discipline of implementation science. We describe five key challenges unique to systematic reviews of primary implementation research; we then provide solutions and highlight resources that can be used by authors of primary implementation research, as well as systematic review and editorial teams, to address the identified challenges. A summary of proposed resources is included in Table 1.

**Table 1:** Overview of resources presented

Resource	Overview
Standards for Reporting Implementation Studies (StaRI) checklist [12]	Guides the transparent and accurate reporting of primary implementation studies Its use enables the clear distinction between evidence-based interventions and implementation strategies, and the identification as an implementation study in an abstract, title and/or keywords
‘Implementation science’ subject heading	Facilitates the identification of implementation science literature within systematic searches
Recommendations for specifying and reporting implementation strategies [13]	Enhances the specification and reporting of implementation strategies
Behaviour change technique (BCT) taxonomy [14]	Utilised to consistently describe implementation strategy components
Expert Recommendations for Implementing Change (ERIC) compilation [15]	Utilised to consistently describe and/or categorise implementation strategies
Framework for Reporting Adaptations and Modifications-Enhanced (FRAME) [16]	Facilitates the tracking and reporting of adaptations and modifications to evidence-based interventions
Framework for Reporting Adaptations and Modifications-Enhanced—Implementation Strategies (FRAME-IS) [17]	Facilitates the tracking and reporting of adaptations and modifications to implementation strategies
TIDieR checklist [18]	Guides the comprehensive reporting of evidence-based interventions
Rating of Included Trials on the Efficacy-effectiveness Spectrum (RITES) [19]	Tool that characterises randomised trials included in a systematic review on an efficacy (explanatory)-effectiveness (pragmatic) continuum
Proctor et al.’s Implementation Outcomes Framework [20]	Specifies and defines implementation outcomes
RE-AIM Framework [21]	Specifies and defines implementation outcomes
Implementation Outcomes Repository https://implementationoutcomerepository.org/	Resource that can assist implementation researchers and stakeholders to search for quantitative implementation outcome instruments

### Challenge 1: descriptors used in implementation science publications

The first major challenge impacting the identification of relevant primary implementation research is inconsistent terminology and sub-optimal indexing in bibliographic databases. Implementation science is inherently interdisciplinary and prior to the formal establishment of the discipline in the early 2000s, primary implementation research articles were published in a wide range of journals across different disciplines [1]. This has contributed to a multitude of terms and definitions used within implementation science that vary across discipline, time period and country (examples include research utilisation, knowledge translation, and dissemination and implementation) [2]. The myriad of descriptors used within publication titles, abstracts and keywords of primary implementation research can greatly affect the ability for systematic review teams to identify publications with an implementation focus. An added layer of complexity is that some studies can be incorrectly classified as reporting on an implementation study, when they report on health outcomes (i.e. clinical effectiveness) rather than implementation processes, strategies, or outcomes. Such identification challenges necessitate broad search criteria, with large numbers of abstracts retrieved. The ensuing lengthy screening processes increase the risk that review findings are out of date or inaccurate by the time a systematic review is published [22].

The aforementioned StaRI checklist [12] is one resource that can partially address these identification challenges. StaRI predominantly suits primary implementation research that evaluates implementation strategies, but some items are applicable to other study designs and/or implementation foci. The 27-item checklist includes a requirement for identification as an implementation study in the abstract, title and/or keywords. Furthermore, as of 2019, ‘*implementation science*’ was introduced as both a Medical Subject Heading (MeSH) and Emtree term. Over time, the appropriate use of this subject heading will facilitate the identification of implementation science literature within systematic searches. It is therefore essential that authors of primary implementation research, as well as systematic review teams, utilise the StaRI checklist and ‘implementation science’ subject heading when reporting and searching for implementation science literature in future research.

### Challenge 2: distinction between evidence-based interventions and implementation strategies

Within the field of implementation science, the evidence-based intervention (i.e. the evidence-based practice, programme, policy, process, or guideline recommendation that is being implemented or de-implemented) is distinct from implementation strategies (i.e. the methods or techniques used to enhance the adoption, implementation and sustainability of an evidence-based intervention) [12]. Unless primary implementation research has been specifically presented for an implementation science audience, researchers often do not differentiate between evidence-based interventions and implementation strategies, with the latter commonly being inconsistently labelled and inadequately described [13]. Furthermore, even when implementation strategies are precisely described using conventional terms, the application and modification of implementation strategies throughout an implementation effort are frequently not tracked or reported in publications. Such challenges limit the ability for systematic review teams to identify relevant literature, synthesise results across studies, and ultimately make conclusions that support the utilisation of specific strategies for a given context.

Published reporting guidelines can assist authors of primary implementation research to comprehensively report on implementation strategies and differentiate them from evidence-based interventions. The StaRI [12] checklist described above is divided into two columns; the first relates to implementation strategies, the second relates to the evidence-based interventions. As the central focus of implementation science is the implementation strategy, it is expected that the first column of the StaRI checklist should always be completed for all primary implementation studies that evaluate implementation strategies [12]. Additional reporting guidelines exist to guide the comprehensive reporting of evidence-based interventions; an example is the Template for Intervention Description and Replication (TIDieR) checklist and guide [18]. While these guidelines are beneficial for the reporting of primary implementation research, it should be noted that more sophisticated standards are still required to guide the comprehensive reporting of systematic reviews of primary implementation research.

Beyond reporting guidelines, a suite of additional resources exists to enhance the specification and reporting of implementation strategies. Proctor, et al. [13] have developed explicit guidance to name, define and operationalise implementation strategies, and taxonomies such as the BCT taxonomy [14] and ERIC compilation [15] — including setting specific adaptations [23, 24], can be used to consistently describe and/or categorise implementation strategies. Furthermore, frameworks and methods have recently been published that can facilitate the tracking and reporting of adaptations and modifications to both evidence-based interventions (e.g. Framework for Reporting Adaptations and Modifications-Enhanced (FRAME) [16]) and implementation strategies (e.g. FRAME-IS [17]). The broad application of such methods within primary implementation research will enable systematic review teams to rigorously assess the impact of evidence-based interventions and implementation strategies.

### Challenge 3: assessment of external validity

Systematic review teams routinely use design-specific critical appraisal tools to assess the methodological quality of a primary study, including risk of bias (e.g. the Cochrane Risk of Bias 2 (RoB 2) tool [25] for randomised controlled trials). Such instruments are concerned with internal validity, which prioritises the establishment of a causal connection between the evidence-based intervention and outcomes (at the patient/population/service level) [26]. While internally valid findings are of value, it is not often feasible or appropriate to utilise methods that would minimise risk of bias in implementation studies, such as blinding of participants and personnel. In addition to internal validity, implementation studies also prioritise external validity, whereby the generalisability of findings to real-world populations and settings is emphasised [26]. A common criticism of systematic reviews in general is that they exclude or inconsistently address external validity in their conclusions [27]. This limits the ability for implementers, such as healthcare practitioners, health service leaders, and policy-makers, to gauge the applicability of review findings to their particular context.

Specific guidance for the reporting of external validity within systematic reviews of implementation studies still requires development within the discipline. A simple classification of external validity (e.g. high, low) may be elusive due to the variety of potential populations, settings, and systems where implementation occurs. However, systematic review teams can report on dimensions of external validity by describing the context of included studies to enhance the interpretation and generalisability of review findings. While the inclusion of contextual information may be limited in the reporting of primary studies, the quality of reporting will likely improve over time due to the availability of guidelines such as the StaRI and TIDieR checklists [12, 18]. When relevant and practical, systematic review teams should attempt to contact corresponding authors to obtain required contextual information, such as characteristics of the reference population (to determine the representativeness of sample population), or characteristics of the service delivery setting (e.g. staffing, resources, urbanicity), though the additional time required to do this should be acknowledged. Editorial boards could potentially assist this process by updating their submission guidelines to encourage authors of primary studies to include contextual information within supplementary files. Another approach that can be utilised by systematic review teams to reflect on external validity is the consideration of how pragmatic or explanatory a primary implementation study is. The RITES (Rating of Included Trials on the Efficacy-effectiveness Spectrum) [19] tool is one example that could be used for studies with a trial design.

### Challenge 4: synthesis of studies with substantial clinical and methodological diversity

Implementation science deals with complexity, and in addition to the terminology and reporting challenges previously noted, methodological and contextual heterogeneity of implementation studies presents challenges for the synthesis of findings. There are a multitude of implementation outcomes, identified in a range of evaluation frameworks [28] that can be assessed across several phases of research and within multi-level systems. Researchers undertaking primary implementation research also utilise a range of study designs, some of which may have no comparison group or use alternative designs such as cluster designs, hybrid designs, stepped-wedge designs, multiple baseline, and controlled before-and-after studies [29]. While meta-analyses are often seen as the ‘gold standard’ for evidence-based decision-making in healthcare, their use may not be possible for systematic reviews of implementation research that seek to synthesise findings from a range of primary studies with diverse characteristics or outcomes [30]. Even when meta-analyses are suitable and performed, it is common for interquartile ranges to be large [31], indicating variability between studies. Therefore, systematic review and editorial teams should not undervalue alternative synthesis methods. The Cochrane Handbook for Systematic Reviews of Interventions has a chapter dedicated to ‘synthesising and presenting findings using other methods’ [30] that can guide systematic review teams in acceptable methods, such as summarising effect estimates and vote counting based on the direction of effect.

### Challenge 5: variability in defining implementation ‘success’

Numerous evaluation frameworks exist within implementation science that can assist authors of primary implementation research to specify and define implementation outcomes (e.g. Proctor et al.’s Implementation Outcomes Framework [20] and The RE-AIM framework [21]). These outcomes (e.g. acceptability, adoption, appropriateness, costs, feasibility, fidelity, penetration, and sustainability) are commonly used as indicators of implementation ‘success’; the challenge for systematic review teams is that implementation outcomes are inconsistently reported on in primary studies, which subsequently impacts on the synthesis of findings within systematic reviews.

Various pragmatic considerations may influence the collection or clear articulation of implementation outcomes within primary implementation research. One example is sustainability, which refers to the “extent to which a newly implemented treatment is maintained or institutionalized within a service setting’s ongoing, stable operations” [20]. This outcome can only be assessed in the longer-term, therefore studies with shorter funding and/or follow-up periods may not collect this data. Additionally, authors of primary implementation studies may have difficulty in addressing both implementation outcomes and health outcomes within strict word limits of journals. Ultimately, a decision to prioritise health outcome reporting may be driven by broader expectations for the need to demonstrate the clinical impact of an evidence-based intervention. The routine use of the StaRI checklist [12] will facilitate the separate reporting of intervention success and implementation success; editorial boards could further assist authors to follow this guidance by adjusting the article word limits for studies with an implementation focus.

An additional challenge when assessing implementation success is that instruments (i.e. tools) used to assess implementation outcomes are unevenly distributed across the key outcomes and largely display unknown psychometric quality. For instance, in a systematic review of implementation outcome instruments, only six out of the 150 identified instruments were related to appropriateness, the majority of which had missing psychometric information [32]. The development of reliable and valid implementation outcome measures is of continued focus within the discipline of implementation science. The Implementation Outcomes Repository (https://implementationoutcomerepository.org/) is a regularly updated resource that can assist implementation stakeholders to search for quantitative implementation outcome instruments.

The identification of implementation mechanisms is another emerging priority within implementation science that moves beyond a sole focus on implementation ‘success’, to additionally explore how and why implementation strategies work. Mechanisms are defined as “processes or events through which implementation strategies operate to affect one or more implementation outcomes” [33]. By understanding mechanisms, it will enable implementation teams to be guided when matching, tailoring, and optimising implementation strategies for specific problems and contexts. At present, the empirical investigation of implementation mechanisms remains under-researched [34]. To advance the discipline and enable systematic review teams to synthesise information about mechanisms, we encourage research teams to focus on mechanisms in the design, conduct and reporting of future implementation studies.

## Conclusion

Consolidation of the literature using systematic reviews is a critical way to progress a field and support evidence-based decision-making in healthcare; however, certain challenges are unique to reviews of implementation science studies. Unless we understand and specify ways to alleviate these issues, the rigour and usefulness of future systematic reviews of implementation science literature will be compromised. In this article, we have reflected on the challenges and potential solutions for conducting systematic reviews in implementation science. The discipline is continually evolving and with it comes the availability of new methods and guidelines. It is essential that implementation researchers continue to develop more sophisticated processes and standards to enhance the value and precision of implementation science review findings for end-users of that knowledge; including healthcare practitioners, health service leaders and policy-makers.

## Data Availability

N/A.
